# Global research landscape and multisystem health mechanisms of luteolin: a comprehensive bibliometric and network pharmacology study

**DOI:** 10.3389/fnut.2026.1758832

**Published:** 2026-01-28

**Authors:** Huina Guo, Xiyan Tian, Haiyan Wang, Kexin Wang, Qi Han, Yujia Guo, Xiaoya Guan, Zhongxun Li, Xin Wen, Bohui Wu, Liting Zhao, Ying Wang, Hongliang Liu, Chunming Zhang

**Affiliations:** 1 Shanxi Key Laboratory of Otorhinolaryngology Head and Neck Cancer, First Hospital of Shanxi Medical University, Taiyuan, China; 2 Shanxi Province Clinical Medical Research Center for Precision Medicine of Head and Neck Cancer, First Hospital of Shanxi Medical University, Taiyuan, China; 3 The First Clinical Medical College of Shanxi Medical University, Taiyuan, China; 4 Department of Ultrasound Medicine, Jinyun People Hospital, Lishui, China; 5 The Basic Medical Sciences College of Shanxi Medical University, Taiyuan, China; 6 Department of Otolaryngology Head & Neck Surgery, First Hospital of Shanxi Medical University, Taiyuan, China

**Keywords:** bibliometric, dietary flavonoid, functional foods, luteolin, multisystem health effects, network pharmacology

## Abstract

**Objective:**

This study employs bibliometric and network pharmacology methods to systematically analyze the development trends, knowledge structure, and potential biological mechanisms of luteolin research from 2000 to 2025, providing insights for its application in nutritional health and disease prevention.

**Methods:**

Based on the Web of Science Core Collection (WoSCC), combined with Bibliometrix, VOSviewer, and citespace, we conducted analyses of publication characteristics, keywords, journals, and co-citation networks. Simultaneously, integrating TM-MC, string, and Cytoscape, we constructed a luteolin target-pathway-disease network and performed core target and KEGG enrichment analyses.

**Results:**

Luteolin research exhibits sustained growth, with a significant increase after 2021. Research focus has expanded from early antioxidant and anti-inflammatory mechanisms to metabolic health, immune regulation, tumor suppression, and multisystem protection. Network pharmacology identified 239 potential targets, with core targets including TP53, TNF, STAT3, and EGFR, significantly enriched in p53, PI3K–Akt, TNF, and IL-17 pathways. Disease association networks indicate luteolin’s potential to intervene in neurological, circulatory, metabolic, immune, digestive, respiratory disorders, and multiple tumors, exhibiting typical multi-target comprehensive regulatory characteristics.

## 1 Introduction

Natural products are regarded as vital resources bestowed upon humanity by nature, whose rich chemical diversity has long inspired novel drug development (1, 2). They serve not only as the starting point for drug discovery but also as a crucial foundation for constructing lead compound structures (3). Currently, approximately 60% of clinically used drugs can be traced back to direct extraction or structural inspiration from natural products. Classic drugs such as aspirin, digoxin, morphine, and artemisinin all originate from natural compounds. Historically, these natural molecules have been used to alleviate symptoms, prevent diseases, and promote recovery from various pathological states (4). Flavonoids, as important natural products, have garnered extensive attention for their remarkable antioxidant, anti-tumor, and anti-inflammatory activities, as well as their ability to regulate multiple key cellular enzymes (5). These properties confer broad application potential in drug development, functional foods, and nutritional health (2).

Luteolin (3′,4′,5,7-tetrahydroxyflavone) is a natural flavonoid compound (2, 6–8), found in numerous plants including many edible species such as celery, broccoli, green peppers, parsley, thyme, dandelion, perilla, chamomile tea, carrots, and olive oil (1). Luteolin exhibits diverse biological activities including cytotoxic effects, anti-inflammatory activity (9), antioxidant activity (10), antitumor activity (11), and antibacterial activity (12, 13), leading to its widespread application in the food industry and biomedical fields (14). Research indicates luteolin is an effective SARS-CoV-2 cell invasion inhibitor (15) and has been recommended as a dietary supplement against COVID-19 (16). Furthermore, Hou et al. (17) demonstrated that luteolin alleviates pulmonary edema in acute respiratory distress syndrome, inhibits inflammatory responses to slow disease progression, and enhances the activity of multiple antioxidant enzymes by regulating oxidative stress levels (18). As related research deepens, luteolin has gradually emerged as a significant research focus in natural drug development and disease prevention. However, the continuous growth in literature volume makes it challenging for researchers to systematically grasp the development trajectory and research frontiers in this field.

**Figure 1:**
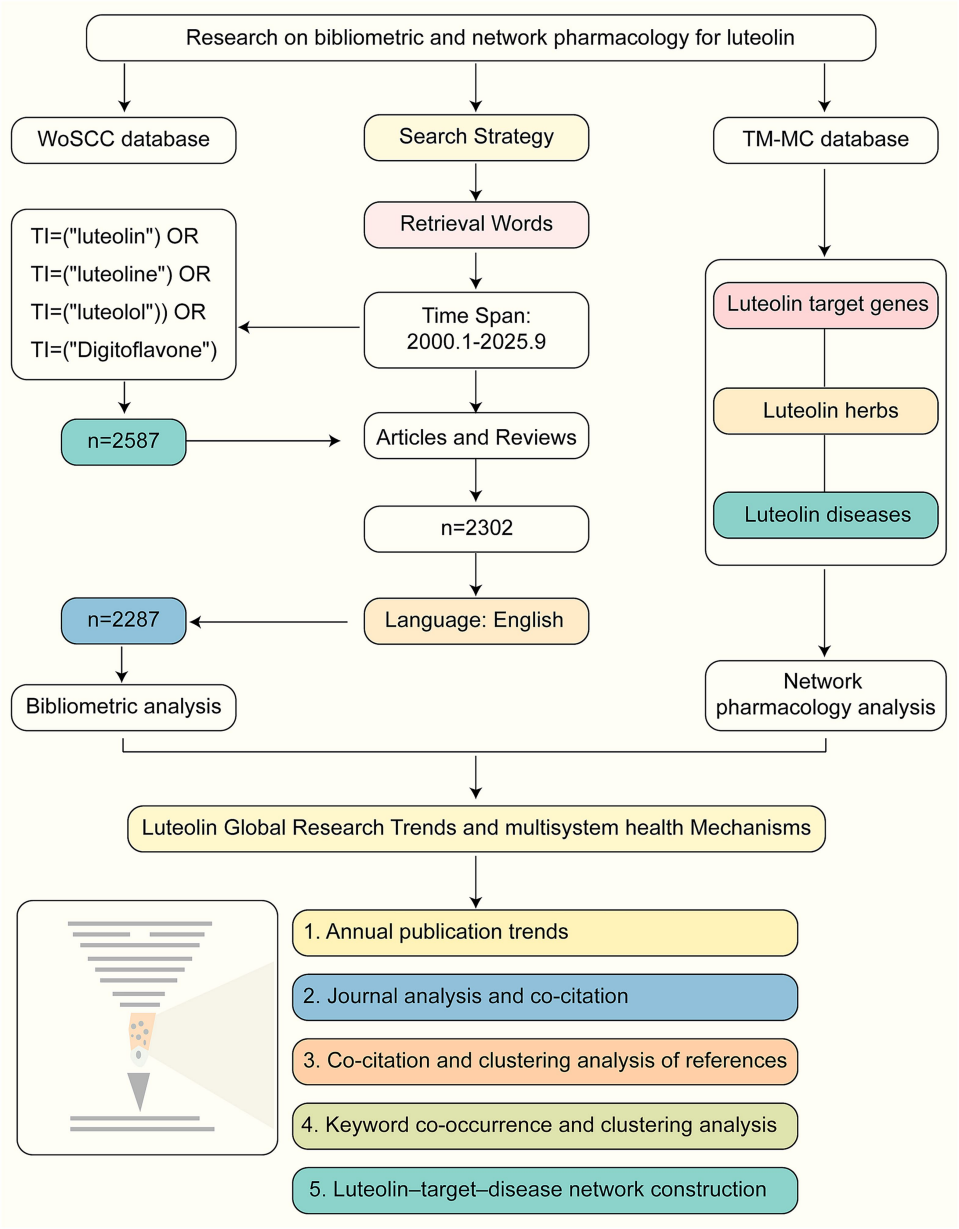
Article strategy and analysis flowchart.

This study systematically reviews evolutionary trends, key target molecules, and associated diseases in luteolin research based on literature from 2000 to 2025 indexed in the WoSCC, integrating bibliometric and network pharmacology analyses. Figure 1 illustrates the data retrieval process and an overall analytical framework. This study aims to comprehensively present the current state and structural characteristics of this field, identify potential research hotspots, and provide references and novel analytical perspectives for future research on luteolin in nutrition and health.

## 2 Materials and methods

### 2.1 Data collection

The WoSCC is a major research platform spanning multiple fields including natural sciences, social sciences, arts, and humanities (19), and one of the most widely cited comprehensive academic literature databases globally (20). The literature extracted for this study originated from the WoSCC database. Searches were conducted using the terms TI = (“luteolin”) OR TI = (“luteoline”) OR TI = (“luteolol”) OR TI = (“Digitoflavone”), with the retrieval timeframe set from January 1, 2000, to September 30, 2025. The initial search yielded 2,587 documents. Subsequently, screening based on document type retained only Articles and Review Articles of high academic and citation values, resulting in 2,302 documents. Further restrictions on English-language documents ultimately included 2,287 documents for subsequent analysis. All documents were exported as complete records containing reference lists and saved as plain text files (download_.txt). It should be noted that due to ongoing updates to the WoSCC database and dynamic indexing status changes, the number of search results may exhibit slight variations. This is when applying the same search strategy at different points in time. The literature search and screening process for this study strictly adhered to the PRISMA 2020 checklist requirements (Supplementary Material S1) (21).

### 2.2 Grey prediction model

This study employs the Grey Model [GM (1.1)] (22, 23) to analyze publication trends and future predictions. To predict publication volumes for the next 3 years using R 4.5.1 software (Supplementary Material S2), a time series forecasting model was constructed using annual publication volume data from 2000 to 2024.

### 2.3 Bibliometric analysis and visualization

Import the articles retrieved in this study into bibliometrix, citespace, and VOSviewer for further bibliometric analysis. The bibliometrix software package captures and extracts relevant information from selected articles, including subject areas, journals, keywords, and country distribution. VOSviewer and citespace are two widely used visualization tools in current bibliometric research, each offering distinct advantages. VOSviewer excels at constructing keyword co-occurrence networks, offering intuitive visualizations that reflect clustering relationships and academic connections through node size, color, and edge thickness (24). Citespace, meanwhile, focuses on revealing the developmental trajectories of research fields and the evolution of research hotspots. Through clustering information and burst term analysis, citespace effectively displays the temporal evolution of research themes, significant turning points within the field, and the citation paths of key publications. The integration of these two software tools facilitates multidimensional exploration of knowledge structures and developmental trends within luteolin research. This provides researchers with comprehensive and systematic theoretical references.

### 2.4 Luteolin-target-disease network construction

This study screened luteolin-related targets, disease information, and herbal sources using the TM-MC database.^1^ Subsequently, the string website^2^ was utilized to construct a protein–protein interaction (PPI) network, aiming to uncover functional connections between the targets. Building upon this, cytoscape 3.6.0 software was employed to construct a luteolin-target-disease network diagram. From this network, key targets potentially involved in luteolin’s biological activity and their primary disease types were identified, providing a reference framework for subsequent research.

## 3 Results

### 3.1 Publication trends and future prediction of luteolin research

In bibliometrics, publication volume serves as a key indicator for measuring research activity (24). Based on the WOSCC, this study included 2,287 publications related to luteolin from January 2000 to September 2025. Statistics show that these articles were published in 840 journals, received 81,401 citations, and generated 43,698 cited references. The H-index stands at 114, with an average citation count of 35.59 per article.

**Figure 2:**
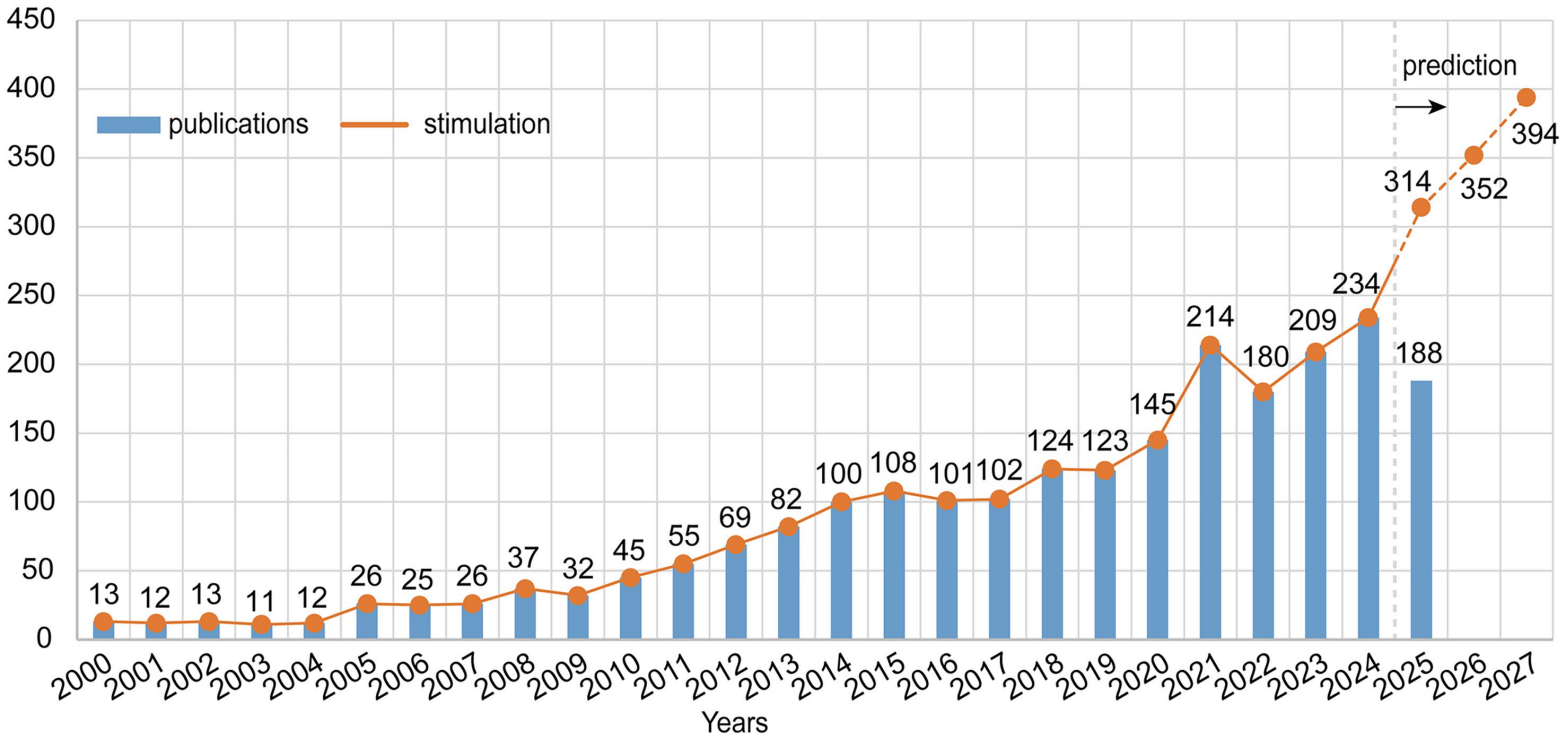
Annual publication trends and future predictions of luteolin.

The annual publication volume shows a steady upward trend in luteolin research from 2000 to 2024 (Figure 2). Early publications were relatively scarce with gradual growth; since 2010, annual output has steadily increased, particularly with a significant surge in 2021. To further explore future trends, this study employed GM(1.1) to predict publication volumes over the next 3 years. Results indicate that luteolin research will maintain an upward trajectory, with projected publications exceeding 390 by 2027. This predicts sustained growth in research activity in this field.

### 3.2 Country and institutional collaboration network analysis

**Figure 3:**
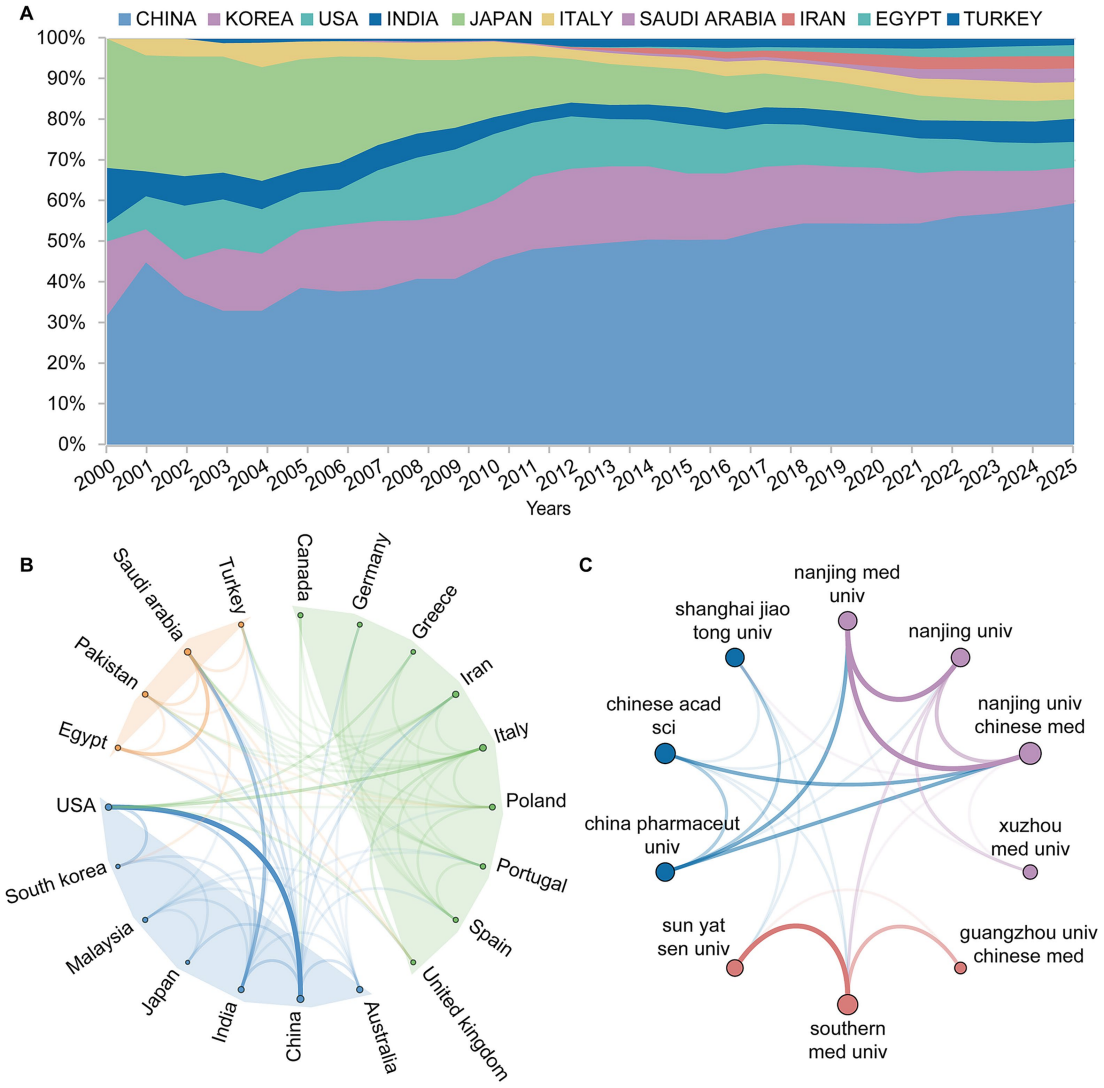
The distribution of collaborative publications and institutions by country. **(A)** Annual proportion of publications on luteolin contributed by major countries from 2000 to 2025. **(B)** Clusters of the country collaboration network. China and USA show the strongest collaboration intensity. **(C)** Institutional collaboration network clusters.

To reveal the global distribution patterns and collaborative characteristics of luteolin research, this study conducted a systematic analysis of publication volumes across major countries. Results indicate that China consistently leads in annual publication output, followed by South Korea and the USA (Figure 3A). In terms of total citation counts, China also ranks first, with South Korea and the USA occupying the second and third positions, respectively (Supplementary Figure S1). Then, this study utilized VOSviewer and Scimago Graphica to visualize collaboration networks among the top 30 countries. The collaborative networks were clustered into three groups, with countries within the same cluster exhibiting close cooperation. China and the USA were the most closely connected countries across different clusters (Figure 3B). The institutional collaboration network (Figure 3C) revealed that Nanjing university of Chinese medicine and the Chinese academy of sciences occupied central positions, each forming close collaborations with multiple institutions.

### 3.3 Journals and co-cited journals analysis

We utilized the Bibliometrix package to identify high-impact journals in the luteolin field and conducted visualization analysis using citespace. Table 1 presents the top 10 journals ranked by publication volume and citation performance. These journals are primarily distributed across JCR Q1 and Q2, with the *Journal of Agricultural* and *Food Chemistry* leading in both publication volume and citation count. Notably, among the top 10 journals by co-citation, high-impact journals such as *Food Chemistry* (IF = 9.8, Q1) and *Cancer Research* (IF = 16.6, Q1) are included.

**Table 1:** The number of publications, IF, and JCR quartile of the top 10 and co-cited journals.

No.	Journal	Publication	IF (JCR2025)	JCR quartile	Co-cited journal	Publication	IF (JCR2025)	JCR quartile
1	J AGR FOOD CHEM	35	6.2	Q1	J AGR FOOD CHEM	1,680	6.2	Q1
2	INT J MOL SCI	33	4.9	Q1	J BIOL CHEM	1,316	3.9	Q2
3	FRONTIERS IN PHARMACOLOGY	31	4.8	Q1	INT J MOL SCI	1,138	4.9	Q1
4	MOLECULES	31	4.6	Q2	PLOS ONE	1,126	2.6	Q2
5	PLOS ONE	30	2.6	Q2	MOLECULES	1,062	4.6	Q2
6	EUROPEAN JOURNAL OF PHARMACOLOGY	27	4.7	Q1	FOOD CHEM	1,001	9.8	Q1
7	BIOMEDICINE & PHARMACOTHERAPY	24	7.5	Q1	BIOMED PHARMACOTHER	950	7.5	Q1
8	INTERNATIONAL IMMUNOPHARMACOLOGY	23	4.7	Q1	J ETHNOPHARMACOL	885	5.4	Q1
9	FOOD & FUNCTION	22	5.4	Q1	CANCER RES	853	16.6	Q1
10	FOOD CHEMISTRY	22	9.8	Q1	P NATL ACAD SCI USA	802	9.1	Q1

**Figure 4:**
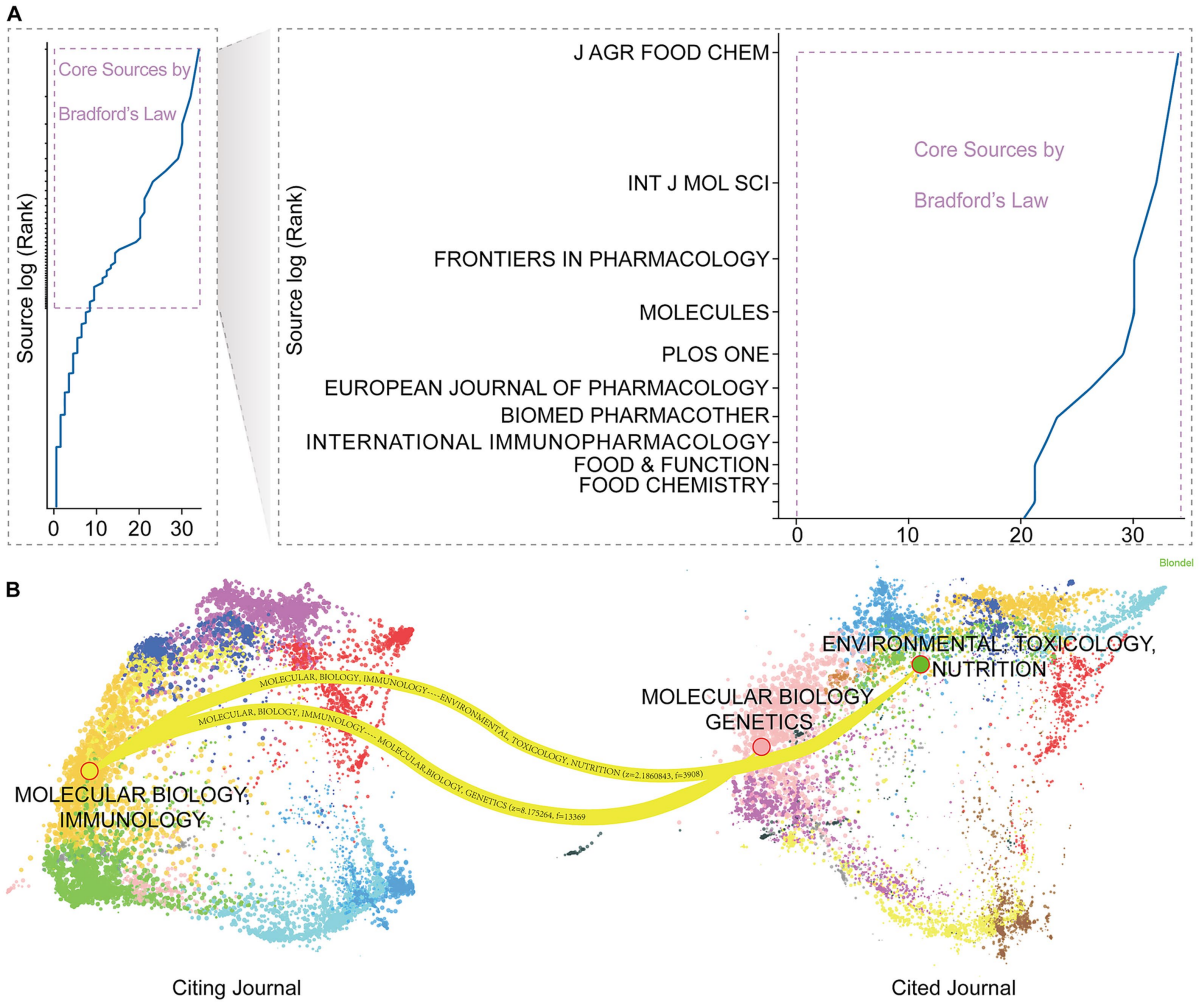
Journal source analysis and dual-map overlay visualization. **(A)** Core journals in luteolin research identified by Bradford’s Law, displaying their distribution and ranking. **(B)** The dual-map overlay visualizes knowledge flow pathways between citing and cited journals.

In addition, Bradford’s Law analysis of journal sources (Figure 4A) reveals that research literature in this field exhibits a “core-related-periphery” distribution pattern. Journals such as *Journal of Agricultural* and *Food Chemistry*, International *Journal of Molecular Sciences*, *Frontiers in Pharmacology*, *Molecules*, and *PLOS ONE* occupy the core zone. They publish the highest volume of articles in this field and serve as primary knowledge output vehicles. Further journal dual-map overlay analysis (Figure 4B) reveals distinct interdisciplinary pathways in luteolin research knowledge flow. Citation sources on the left primarily focus on foundational disciplines like molecular biology/immunology, while cited works on the right concentrate on applied fields such as environmental toxicology and nutrition. These connecting pathways indicate a research shift from fundamental mechanism studies toward nutrition, toxicology, and related health effects.

### 3.4 Co-citation and cluster analysis of literature

To identify the knowledge base and core research trajectories within the luteolin field, this study conducted a co-citation analysis of the literature. Co-citation relationships reveal underlying logical connections between different publications, aiding in clarifying the theoretical underpinnings and developmental trends of research.

**Figure 5:**
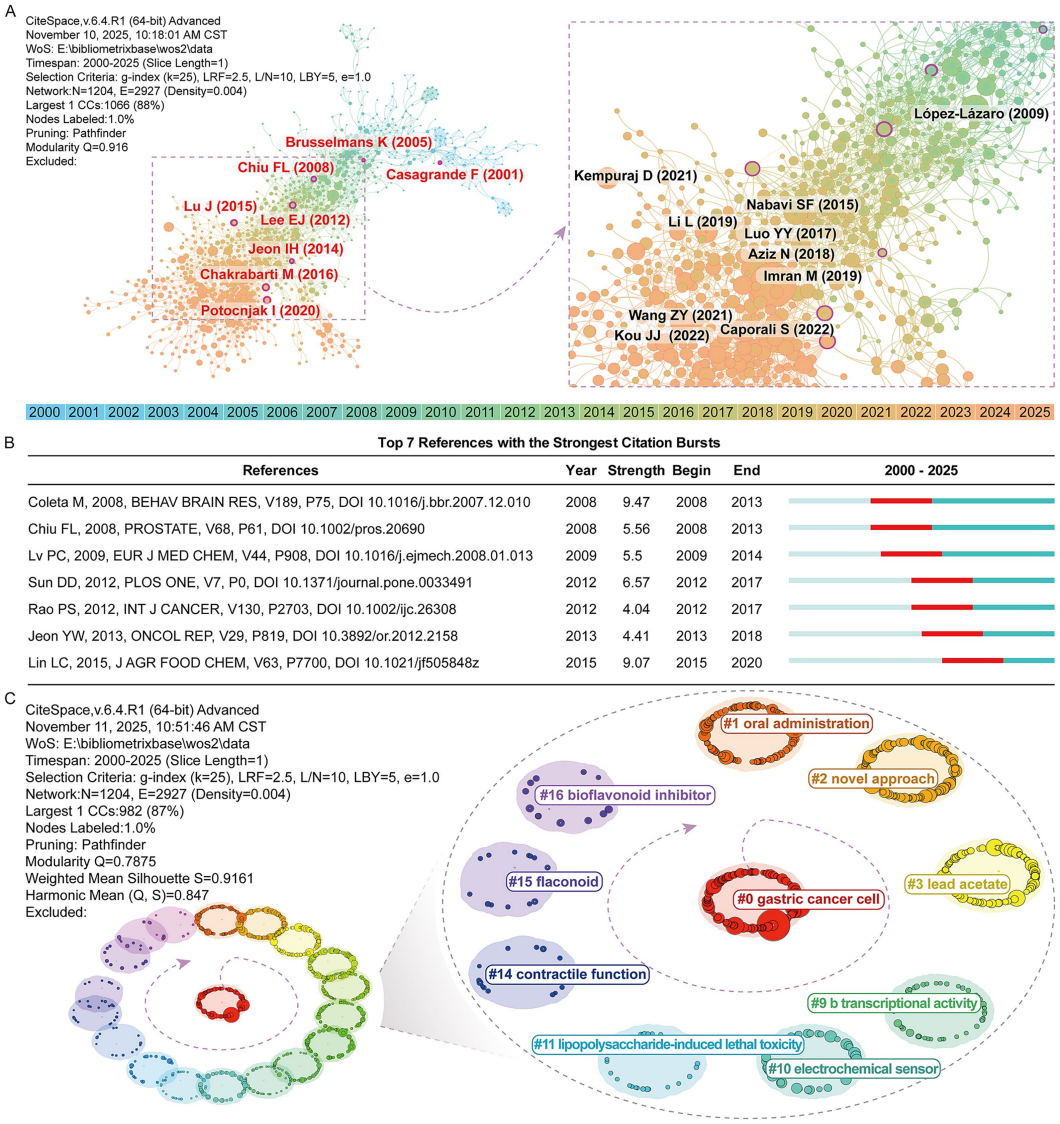
Literature co-citation and cluster analysis. **(A)** Literature co-citation reference network. Nodes with high betweenness centrality values are marked with purple outer rings. **(B)** Top seven references with the strongest citation bursts between 2000 and 2025. **(C)** Thematic evolution map of luteolin-related research based on literature co-occurrence clustering.

Figure 5A displays the top 10 most cited articles and constructs their co-citation network using citespace, with nodes possessing high betweenness centrality values marked by purple outer rings. Additionally, Figure 5B highlights seven articles exhibiting significant citation bursts related to luteolin when the minimum duration was set to 6. These articles were clustered into 18 groups (Figure 5C), with the most prominent being #0 gastric cancer cell. Clusters such as #1 oral administration, #2 novel approach, and #3 lead acetate pertain to formulation innovation, toxicological assessment, and administration methods. In contrast, earlier clusters like “#16 bioflavonoid inhibitor,” “#15 flavonoid,” and “#14 contractile function” indicate that luteolin’s traditional pharmacological effects, structural studies, and chemical mechanisms remain crucial components of the knowledge base.

### 3.5 Keywords co-occurrence and clustering analysis

**Figure 6:**
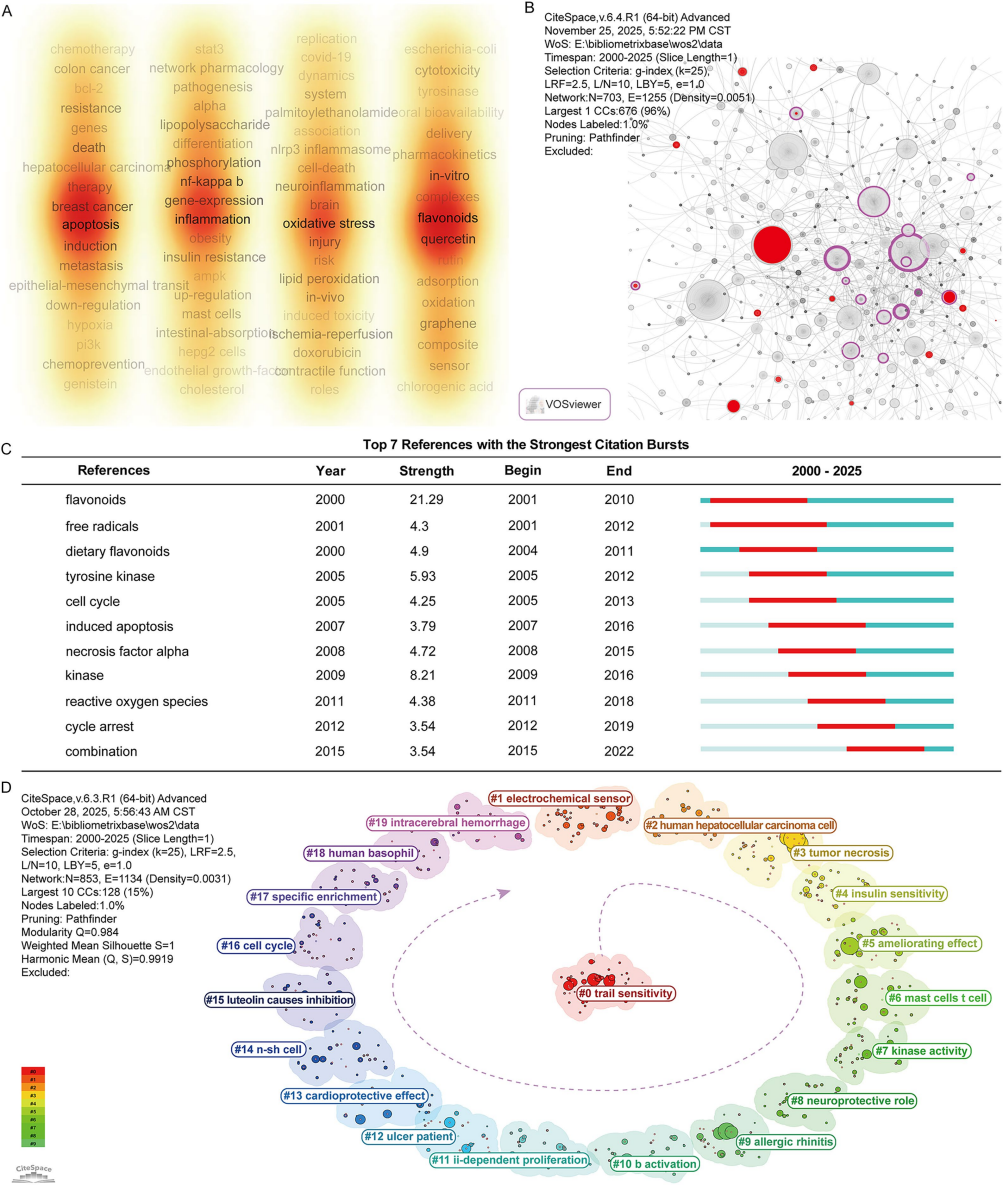
Heatmap of research themes, keyword burst analysis, and keyword clustering in luteolin studies. **(A)** Keyword co-occurrence heatmap highlighting major research themes in the luteolin field. **(B)** Reference co-citation network. Red nodes represent references with strong citation bursts, purple nodes denote high betweenness centrality references. **(C)** The panel shows the top7 references with the strongest citation bursts. **(D)** Keyword clustering network showing major thematic clusters.

Keyword co-occurrence analysis helps reveal connections between different research topics, identifying core research hotspots and potential mechanisms in the luteolin field. This study visualized author keywords using VOSviewer, setting the minimum number of occurrences to 5. After excluding irrelevant or meaningless keywords such as ‘luteolin’, ‘cells’, “expression,” ‘I’, and ‘assay’, a total of 212 keywords were obtained. Figure 6A shows distinct high-frequency clusters. Keywords such as apoptosis, inflammation, oxidative stress, and flavonoids exhibit elevated co-occurrence frequencies and brightness, indicating their centrality in the research landscape of luteolin.

We further performed burst analyses to identify keyword bursts related to luteolin research. The results (Figures 6B,C) show that keyword bursts in the early stages primarily included flavonoids, free radicals, and dietary flavonoids, reflecting a focus on nutritional antioxidant mechanisms and free radical scavenging. Subsequently, terms such as tyrosine kinase, cell cycle, induced apoptosis, necrosis factor alpha, and kinase appeared. These terms indicated a gradual shift in research emphasis toward molecular signaling pathway regulation and cellular-level mechanisms. In recent years, keywords such as reactive oxygen species, cycle arrest, and combination reflect a growing research focus on luteolin’s role in oxidative stress, cell cycle arrest, and multi-component combination applications.

Keyword cluster analysis results indicate that luteolin related research focuses primarily on themes such as antitumor effects, inflammation and immune regulation, metabolic improvement, and cardiovascular and neuroprotection (Figure 6D). The largest cluster, #0 trail sensitivity, represents research hotspots for inducing tumor cell apoptosis; clusters such as #7 kinase activity and #16 cell cycle suggest that molecular mechanism studies remain predominant. Simultaneously, clusters such as #4 insulin sensitivity, #13 cardioprotective effect, and #8 neuroprotective role reflect the gradual expansion of luteolin research into nutrition and chronic disease prevention.

### 3.6 Luteolin target and signaling pathway enrichment

Based on bibliometric analysis, we can clearly observe that luteolin has garnered extensive attention in fields such as anti-inflammatory effects, anti-tumor activity, and immunomodulation, occupying a significant position in research into various diseases. However, as a naturally occurring flavonoid widely present in food sources, luteolin’s health benefits often depend on its comprehensive regulation through multiple targets and pathways. Its underlying mechanisms still require systematic elucidation.

**Figure 7:**
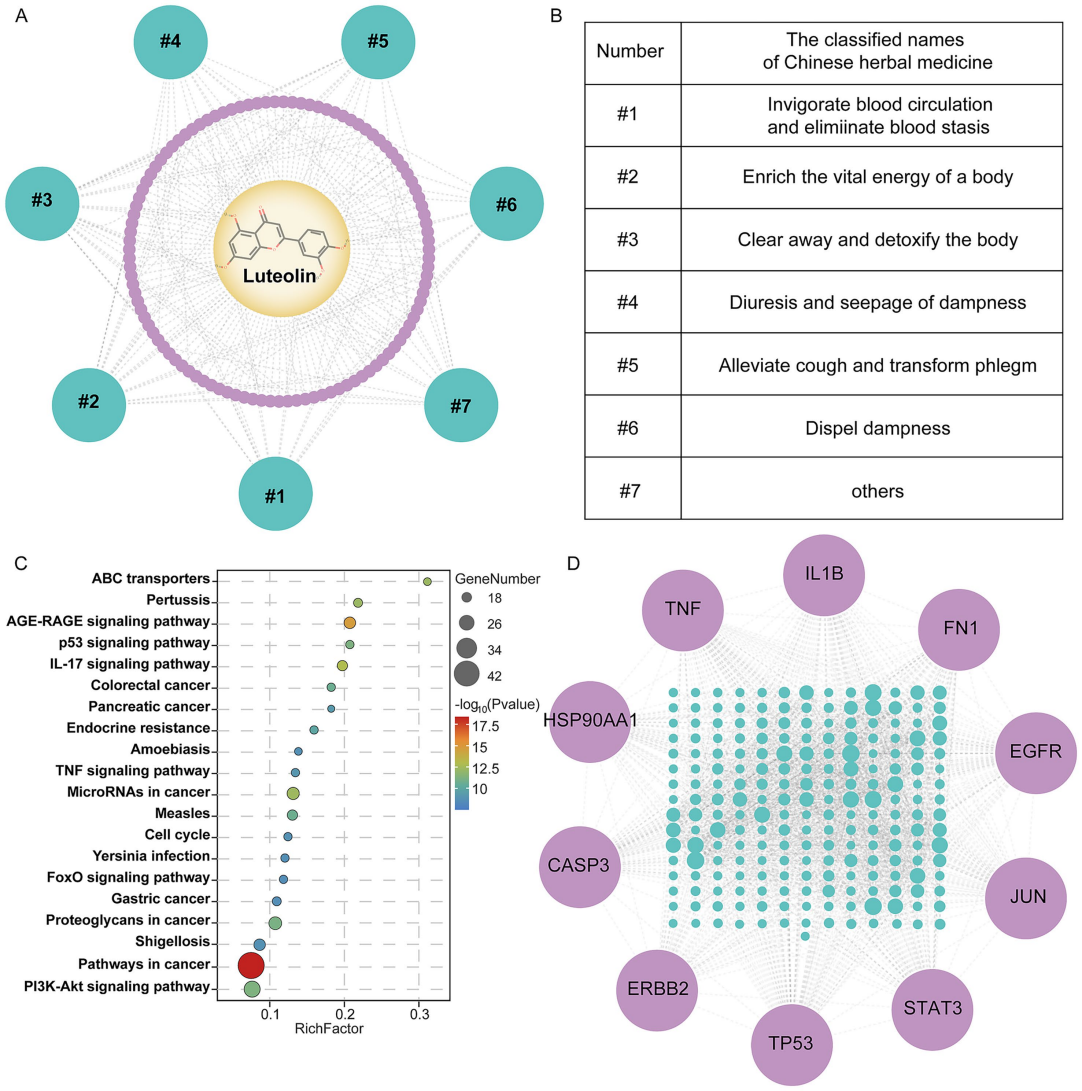
Network pharmacology analysis of the multi-target mechanisms and key signaling pathway enrichment of luteolin. **(A,B)** Distribution of luteolin-containing herbs by efficacy category. Yellow represents luteolin, purple represents herbs, green represents herbs efficacy classification. **(C)** Luteolin target-associated KEGG signaling pathway enrichment. **(D)** PPI network map of luteolin’s targets. Green represents luteolin targets and purple represents 10 core targets.

In order to understand luteolin’s biological actions, this study constructed a network pharmacology model to analyze its sources, targets, and potential functional pathways. First, the TM-MC database identified 113 plant sources containing luteolin (Supplementary Table S1), covering traditional efficacy categories such as clearing heat and detoxifying, promoting blood circulation and removing blood stasis, and dispelling wind-dampness (Figures 7A,B). This also suggested diverse dietary intake pathways for luteolin.

Further screening identified 239 potential targets associated with luteolin (Supplementary Table S2). After constructing a PPI network using the string platform and performing KEGG enrichment analysis, the top 20 pathways were selected based on the false discovery rate (Figure 7C). Results revealed multiple pathways associated with tumorigenesis and progression, significantly enriched in key biological processes including inflammatory regulation, immune control, and metabolic homeostasis—particularly signaling pathways such as p53, PI3K–Akt, IL-17, and TNF. These pathways play central roles in nutrition-related diseases characterized by chronic inflammation, immune dysregulation, and metabolic disorders.

We conducted a topological parameter analysis of the compound-target interaction network by cytoscape software. Employing degree as the primary screening criterion, we identified the top 10 core targets ranked by degree value: TP53, TNF, IL1B, STAT3, EGFR, JUN, CASP3, FN1, HSP90AA1, and ERBB2 (Figure 7D). These targets participate in critical pathways such as the inflammatory response, cell cycle regulation, apoptosis, and cellular stress responses. This suggests that luteolin may exert synergistic biological regulatory effects in nutrition-related diseases by modulating multiple signaling axes.

In summary, luteolin modulates inflammation, immune responses, and metabolic processes through coordinated regulation of multiple molecular targets and signaling pathways, providing theoretical support for its application in nutritional interventions, disease prevention, and functional food development.

### 3.7 Network analysis of diseases associated with core luteolin targets

**Figure 8:**
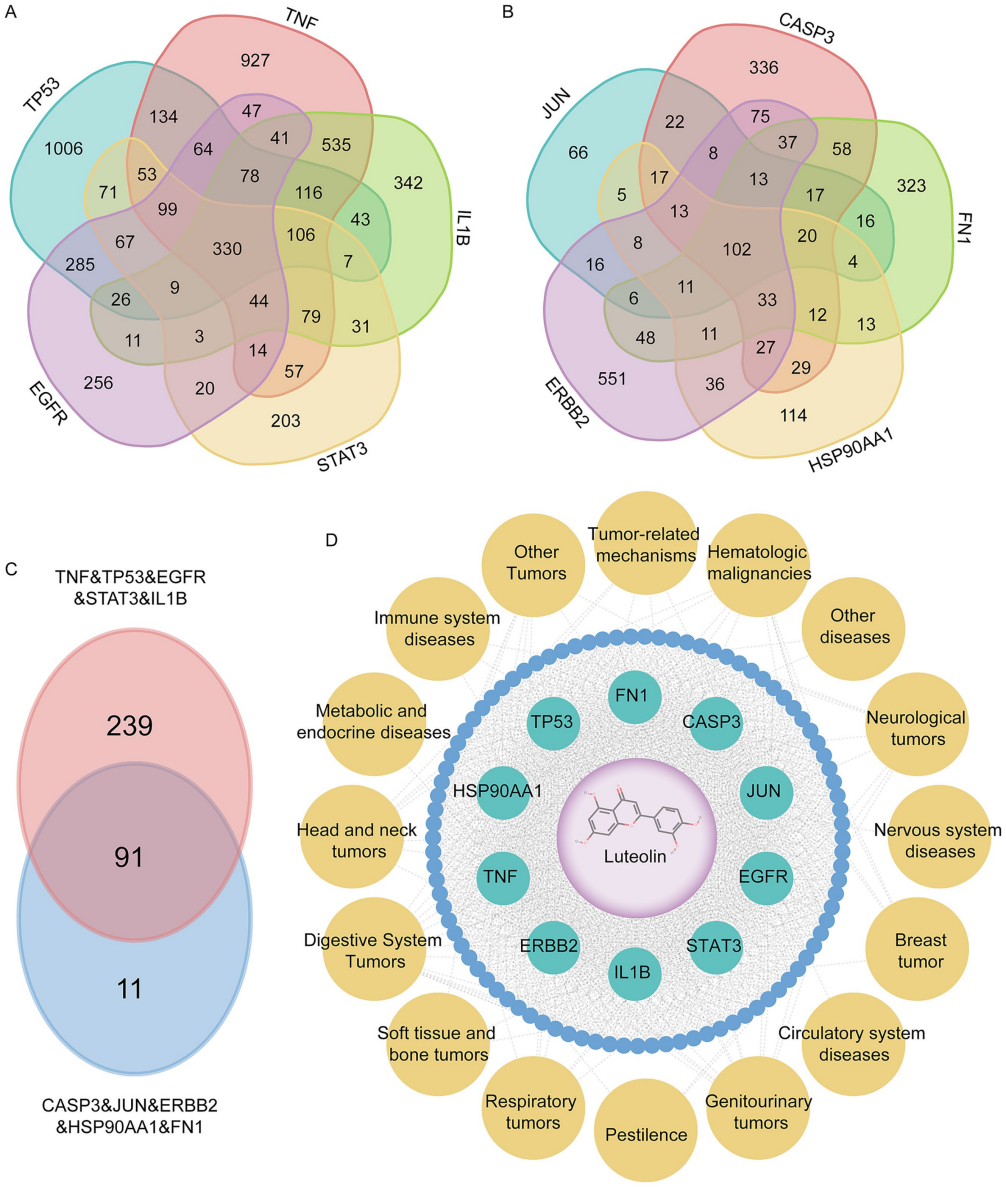
Network construction and classification analysis of diseases associated with core targets of Luteolin. **(A–C)** 10 core target-associated diseases Venn analysis. **(A)** TNF, TP53, IL-1B, STAT3, and EGFR associated diseases Venn analysis acquired 330 diseases. **(B)** CASP3, FN1, HSP90AA1, ERBB2, and JUN associated diseases Venn analysis acquired 102 diseases. **(C)** 330 diseases and 102 diseases Venn analysis acquired 91 diseases. **(D)** Luteolin-core target-diseases network map. Purple represents luteolin, green represents core target, blue represents diseases, yellow represents diseases classification.

Based on the TM-MC database, we identified the top 10 target-related diseases and obtained 91 common diseases through intersection analysis (Figures 8A–C). Subsequently, we systematically categorized disease types and constructed a luteolin-cored target–disease association network (Figure 8D and Supplementary Table S3). Results revealed widespread distribution across neurological, circulatory, respiratory, digestive, immune, and metabolic disorders, alongside multiple tumor types (e.g., breast cancer, head and neck tumors, bone and soft tissue tumors). This pattern suggests that luteolin may exert cross-systemic anti-inflammatory, anti-tumor, immunomodulatory, and tissue-protective effects by acting on diverse molecular nodes involved in key biological processes.

As a whole, luteolin’s broad coverage across diseases and the concentrated distribution of its core targets in key biological processes reflect its potential value as a natural dietary flavonoid for intervening in multisystem diseases. This network characteristic not only supports its potential application to promoting nutritional health and preventing chronic diseases. It also provides crucial evidence for subsequent mechanism studies and multi-indication drug development.

## 4 Discussion

This study comprehensively reveals the knowledge structure, research hotspots, and future development trends of luteolin research from 2000 to 2025 through systematic bibliometric and network pharmacology analyses. Overall, luteolin research exhibits sustained growth, with expanding research themes and significantly deepening mechanistic investigations. Its dual status as both a food and a medicinal substance grants it unique advantages in nutrition science, functional foods, and chronic disease prevention. This aligns closely with recent trends in nutritional health research emphasizing natural bioactive compounds, diet interventions, and precision nutrition.

### 4.1 Luteolin research on the rise: extending from basic pharmacology to nutritional science

Luteolin, as a vital dietary compound, has seen a sustained increase in the number of published papers, indicating growing recognition of its potential value in nutrition, metabolic health, and chronic disease prevention. The accelerated growth in publications since 2010, particularly the significant surge in 2021, may be closely linked to the recent research boom on natural bioactive substances and food-medicine dual-use substances for health promotion. Studies indicate that oral luteolin administration reduces bone loss in ovariectomized rats (25). In Wistar rats, oral luteolin protects the kidneys from lead acetate-induced nephrotoxicity through antioxidant, anti-inflammatory, and anti-apoptotic mechanisms (26). Furthermore, luteolin has been demonstrated to alleviate mucosal tissue damage caused by enterocolitis during cancer treatment (27). The gray prediction model predicts a sustained rapid upward trend in this field over the next 3 years. This predicts sustained research interest in luteolin and highlights its long-term potential in nutrition and health sciences.

### 4.2 Research themes have expanded from fundamental mechanisms to multisystem health effects

Journal and co-citation analysis suggests that core journals for luteolin research are predominantly concentrated in the food science and life sciences, maintaining substantial academic influence. This aligns with existing studies emphasizing luteolin’s prominence in food chemistry, pharmacology, and biomedicine (28, 29). The co-citation structure further revealed a distinctly multidisciplinary research foundation for luteolin. Mechanistic studies on its anti-inflammatory effects (30), immunomodulation (31), and antitumor activity (32) have reached relative maturity, providing robust molecular-level evidence for its biological activity. Bradford’s zoning analysis suggests that journals in food science and nutrition constitute the core knowledge output platforms in this field. This reflects a transition in luteolin research from basic exploration at the single-compound level toward application-oriented nutritional science (31).

Meanwhile, the knowledge flow pathways revealed by the journal dual-map overlay indicate that the research focus is expanding beyond traditional molecular biology and immunology into more applied fields such as nutrition, metabolic health, and environmental toxicology. Existing studies demonstrate that luteolin can improve insulin sensitivity, regulate lipid metabolism, and restore energy homeostasis (33). Furthermore, it plays a significant role in protecting the intestinal barrier (34), reducing food-induced oxidative damage (35), and alleviating toxin-induced inflammation (36). Overall, luteolin research has entered a critical phase of transitioning from fundamental mechanisms to multi-system health effects. It is increasingly recognized as a key dietary bioactive compound with potential for nutritional intervention. This lays a solid scientific foundation for functional food development, prevention of inflammation-related diseases, and nutritional regulation of metabolic disorders.

### 4.3 Deepening of molecular mechanism research: inflammation, apoptosis, and metabolic regulation as key axes

Keyword co-occurrence and burst analyses indicate that apoptosis, oxidative stress, and inflammation remain core mechanisms in luteolin research, consistent with extensive *in vivo* and *in vitro* evidence (37). Research indicates that luteolin may exerts synergistic anti-inflammatory and cytoprotective effects by regulating multiple key signaling pathways, including AMPK-PPARγ (38), IL-33-ST2 (39), and TLR4/NF-κB (40), thereby improving immune responses, inflammatory progression and related functional barriers (41). A study indicates that luteolin prevents colon cancer development by inhibiting macrophage polarization toward the M1 phenotype. Specifically, it suppresses M1 macrophage polarization by acting on the IL-6/STAT3 signaling pathway, thereby inhibiting the proliferation, migration, and invasive capabilities of colon cancer cells (42). Further research indicates that luteolin significantly enhances mitochondrial membrane potential depolarization in bladder cancer cells. The promotes the release of cytochrome C and SMAC/DIABLO from the mitochondria into the cytoplasm. This activates the mitochondrial-mediated intrinsic apoptosis pathway. Concurrently, it markedly downregulates the expression of anti-apoptotic proteins Bcl-xl and Mcl-1 while inducing endoplasmic reticulum stress responses, ultimately suppressing bladder cancer cell activity and promoting apoptosis (43). These consistently emerging keywords not only suggest a high degree of consensus among researchers regarding fundamental molecular mechanisms but also reinforce luteolin’s potential value as a dietary flavonoid in preventing nutrition-related diseases and promoting health.

Notably, recent keyword bursts of terms such as reactive oxygen species, cycle arrest, and combinations in recent years indicate that research is increasingly extending toward more refined regulation of cellular fate and information networks. Existing studies demonstrate that luteolin can induce cell cycle arrest by modulating key nodes including CDK2, p21, and p53 (44, 45), thereby inhibiting abnormally proliferating cells, including tumor cells and inflammation-associated cell subpopulations. Concurrently, the potential synergistic effects of luteolin combined with other drugs or nutritional factors in metabolic disorders and tumor therapy are gaining increasing attention (46–48).

Cluster analysis further highlights this trend. For instance, the “#0 TRAIL sensitivity,” “#7 kinase activity,” and “#16 cell cycle” clusters all point to anti-tumor mechanisms involving apoptosis regulation, TRAIL pathway activation, and protein kinase signaling networks. Luteolin enhances pancreatic cancer cells’ sensitivity to TRAIL-mediated apoptosis and inhibits cell proliferation by downregulating miR-301-3p expression in pancreatic ductal adenocarcinoma cells, thereby upregulating its target gene caspase-8 (49). Wu et al. (50) further demonstrated that luteolin enhances non-small cell lung cancer sensitivity to TRAIL-induced apoptosis by upregulating death receptor DR5, inducing Drp1-dependent mitochondrial fission, and activating the JNK signaling pathway. These findings collectively highlight luteolin’s significant role in anti-tumor nutritional research.

Meanwhile, clusters such as “#4 insulin sensitivity,” “#13 cardioprotective effect,” and “#8 neuroprotective role” highlight its potential for nutrition-related chronic diseases. Evidence suggests that luteolin improves insulin signaling pathways, reduces myocardial oxidative damage, and modulates neuroinflammation, thereby demonstrating significant effects on metabolic syndrome (51), cardiovascular diseases, and neurodegenerative disorders (52). Huang’s research indicates that luteolin improves metabolic disorders and alleviates diabetic nephropathy progression by inhibiting NLRP3-TGF-β-mediated inflammatory and fibrotic signaling through AMPK (51). Furthermore, luteolin effectively ameliorates cognitive deficits in Alzheimer’s disease by suppressing Aβ-induced oxidative stress through a PPARγ-dependent mechanism, thereby repairing mitochondrial damage and reducing neuronal apoptosis (10). These findings are indicative of a gradual expansion of luteolin’s sphere of action from localized cellular effects to broader systemic regulation, paralleling the current research shift from “single-target pharmacology” to “multisystem nutritional modulation.”

In general, molecular mechanisms of luteolin are being studied more deeply from classical anti-inflammatory pathways to integrated signaling pathways and systemic metabolic improvement. This trend not only reinforces its biological value as a dietary flavonoid for health promotion. It also provides a solid theoretical foundation for its application in nutritional interventions for chronic diseases.

### 4.4 Multi-target, multi-pathway, multi-system effects reveal the nutraceutical essence of luteolin

Network pharmacology analysis further reveals that luteolin exhibits typical nutraceutical characteristics, characterized by extensive target coverage and coordinated regulation across multiple signaling networks, thereby conferring robust systemic modulatory capacity. For instance, in the mechanism of luteolin’s anti-non-small cell lung cancer activity, network pharmacology analysis identified 47 potential targets, including core genes such as TP53, EGFR, AKT1, and TNF (53). Furthermore, in an osteoporosis model, combined network pharmacology and experimental validation studies revealed that luteolin targets TP53, AKT1, STAT3, and others, regulating signaling pathways such as PI3K–Akt, TNF, and p53 to promote osteogenesis and inhibit bone resorption (25). Another study on dry age-related macular degeneration revealed that through protein–protein interaction network analysis, TP53, TNF, STAT3, IL6, and others were identified as key targets for luteolin, supporting its role in anti-inflammation, anti-stress, and metabolic homeostasis (54). In oncology, a network pharmacology and transcriptomics study on hepatocellular carcinoma identified luteolin-regulated targets associated with inflammation, cell migration, cell cycle, and apoptosis, involving pathways such as EGFR, STAT3, and TP53 (55).

It is noteworthy that although the core targets identified by network pharmacology analysis share certain commonalities across multiple tumors, their specific modes of action may exhibit variability depending on the molecular context of the tumor. In non-small cell lung cancer driven primarily by EGFR, luteolin can inhibit cancer cell progression by targeting the HGF-MET-AKT pathway (56). Conversely, in glioblastoma, luteolin combined with erlotinib binds to the ATP-binding site of the EGFR receptor to suppress its expression, thereby reducing the phosphorylation levels of downstream molecules AKT, NF-κB, and STAT3, effectively inhibiting malignant cell proliferation (57). In contrast, within the context of inflammation-associated tumors such as colorectal cancer, luteolin not only improves the tumor microenvironment by inhibiting pro-inflammatory signaling pathways and modulating T-cell and macrophage function, but also induces ferroptosis by targeting GPX4, thereby exerting unique anti-cancer effects (13, 32). Furthermore, in tumors with frequent TP53 mutations, luteolin assembles in structural clefts in the TP53 Y220C mutation region. This stabilizes the binding domain of mutant TP53 Y220C and restores it to wild-type levels (58).

From an immunoregulatory perspective, luteolin alleviates inflammatory responses in acute pneumonia and colitis by targeting pro-inflammatory factors such as IL-1β, IL-6, and TNF. It accomplishes this by inhibiting the EGFR/MAPK/PI3K-AKT and NF-κB signaling pathways, while inducing macrophage polarization from M1 to M2 (9, 30). Lu et al. (59) further demonstrated that luteolin effectively mitigates TNF-α-induced inflammation damage in human microvascular endothelial cells via the Akt/MAPK/NF-κB pathway. Similarly, the STAT3 and IL-17 signaling axes play pivotal regulatory roles in macrophage inflammatory responses and M1/M2 polarization (59, 60), and are beneficial for regulating T cell subset imbalances (61) and maintaining Treg-related immune tolerance states (62). This comprehensive regulatory mechanism governs immune cell function and immune cytokine production. It provides substantial theoretical support for the potential application of luteolin in nutrition-immunity-related diseases such as inflammation-related conditions.

Furthermore, from a bibliometric perspective, luteolin research keywords have evolved from early macro-level descriptions such as “antioxidant” and “free radicals” toward mechanism-oriented terms like “apoptosis,” “inflammation,” and “kinase activity.” This shift reflects the deepening research paradigm within the field, transitioning from macro-functional observations toward molecular mechanism analysis. Neurodegenerative diseases trigger oxidative stress and inflammation triggered by ROS accumulation. Instead, they are amplified through the activation of a cascade of MAPK/JNK, PI3K–Akt, and NF-κB kinase signaling pathways, thereby regulating neuroinflammation, programmed cell death, and protein homeostasis imbalances (52). Consequently, the earlier research focused on ‘free radicals’ and antioxidant imbalances may be regarded as a phenotypic description of upstream stress signals. In contrast, current studies centered on kinase activity and signaling networks reveal downstream molecular regulatory essence.

Collectively, these findings highlight the recurrent involvement of core nodes including TP53, TNF, IL1B/IL6, STAT3, and EGFR across diverse disease models, underscoring their central roles within luteolin regulated signaling networks. This suggests their pivotal roles in luteolin’s regulation of chronic inflammation, metabolic imbalance, and tumorigenesis. Additionally, pathway analysis encompasses the p53 pathway (63), the PI3K–Akt pathway (64, 65), and the TNF signaling pathway (59). These pathways are closely associated with inflammation, immunity, apoptosis, and metabolic homeostasis (11, 66, 67).

Disease network analysis further suggests that luteolin may potentially intervene in neurological disorders, circulatory system diseases, digestive system diseases, respiratory system diseases, and multiple tumor types, highlighting its potential as a nutritional compound capable of coordinated regulation across multiple biological systems. Collectively, these findings support luteolin’s nutraceutical properties.

### 4.5 Application prospects: from functional nutrition to advanced delivery and synergistic strategies

Integrated results from bibliometrics and network pharmacology, future research and applications on luteolin exhibit multidimensional expansion trends. Its widespread presence in dietary sources and prominent anti-inflammatory and antioxidant activities make it an ideal natural ingredient for functional food and dietary supplement development (68). Regarding chronic inflammation and metabolic-related diseases, network pharmacology analysis reveals luteolin’s broad regulatory capacity across multiple molecular pathways in enhancing insulin sensitivity, regulating lipid metabolism, and alleviating inflammation, demonstrating its significant potential as a nutritional intervention strategy for diabetes and metabolic syndrome (35). Furthermore, luteolin’s strong association with apoptosis and cell cycle arrest targets offers novel directions for nutritional regulation and adjuvant therapy in tumorigenesis and progression (69). Concurrently, the latest keyword evolution and disease association networks indicate rising research interest in luteolin’s role in neurodegenerative diseases and cardiovascular health (70).

Despite extensive research revealing luteolin’s multifaceted biological actions in the anti-inflammatory, immunomodulatory, and anti-tumor contexts, its clinical translation remains constrained by inadequate bioavailability. Luteolin’s poor water solubility, low oral absorption rate, and limited *in vivo* stability prevent it from achieving effective concentrations comparable to those observed in experimental settings (71). This significantly constrains its potential for nutritional intervention, co-creation and translation. Furthermore, current mechanistic studies on luteolin focus on cellular models or animal experiments. These studies have dosage ranges that differ from human dietary intake or functional food applications. This underscores that isolated target and pathway predictions alone cannot substantiate their clinical or nutritional value. Consequently, there is an urgent need to integrate delivery systems and formulation optimization to bridge the gap between mechanistic research and practice application. Notably, synergistic and composite delivery systems are emerging as a key trend to enhance its bioavailability and therapeutic efficacy. This includes constructing nanocomposites with multiple drugs or materials (72), hydrogel delivery systems (73), and synergistic antitumor effects with paclitaxel (74). These approaches open broader avenues for its deepening application in precision nutrition and nutritional therapy.

### 4.6 Limitations and future prospects

Although this study is relatively comprehensive, certain limitations remain: the bibliometric findings are constrained by database scope and may omit some grey literature; the network pharmacology analysis is predictive in nature, and its results require validation through additional *in vitro* and *in vivo* experiments; furthermore, this study has not yet conducted an in-depth exploration of key nutritional evaluation issues such as dose–response relationships, safety, and bioavailability—all of which represent crucial directions for future translational research on luteolin. Looking ahead, future research should integrate clinical nutrition, gut microbiome studies, metabolomics, and precision nutrition models to elucidate luteolin’s biological mechanisms and optimize dosage strategies at the system level. This will advance its practical application to functional food development and nutritional interventions for chronic diseases.

## 5 Conclusion

**Figure 9:**
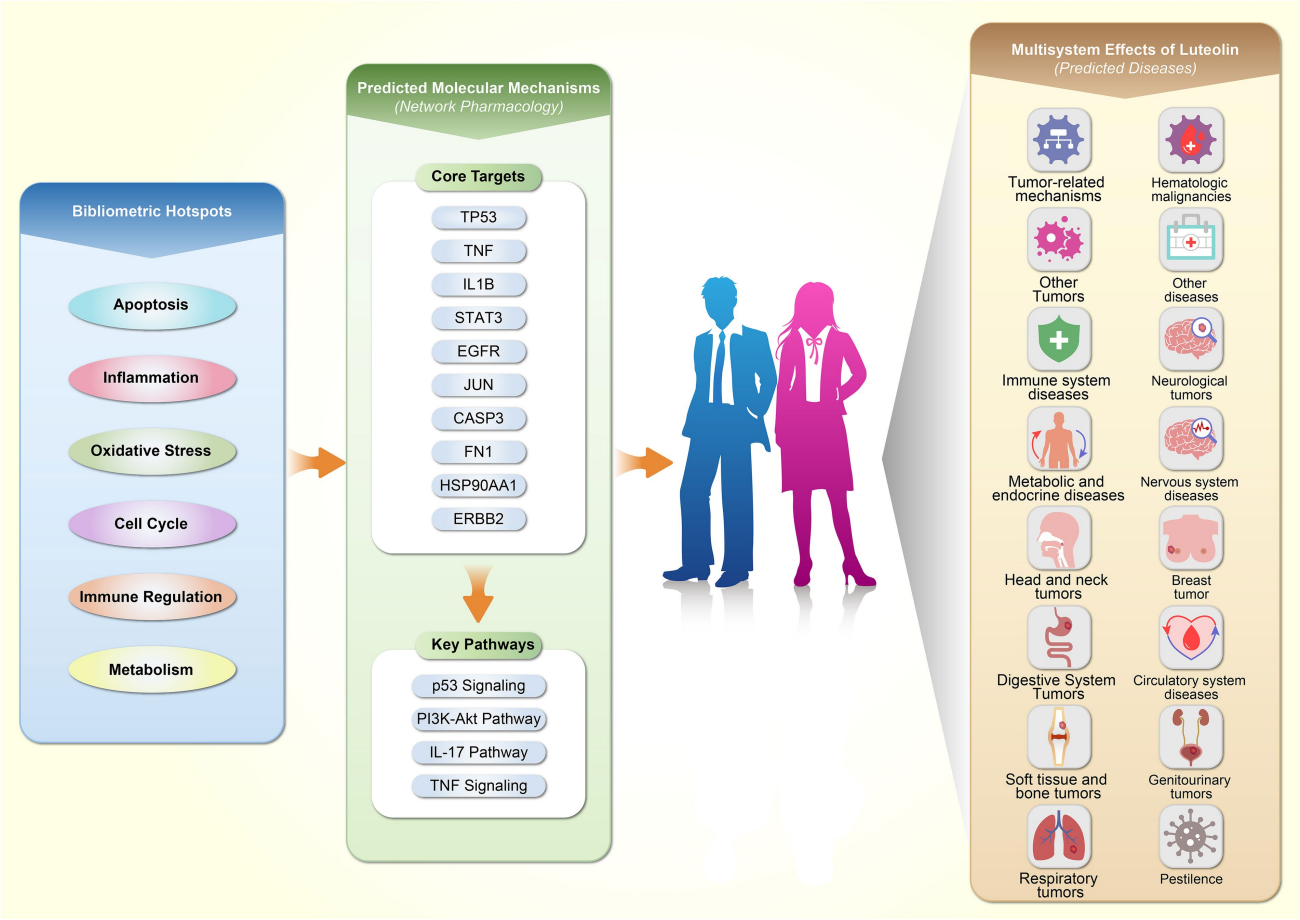
Conceptual model of potential mechanisms of action of luteolin.

This study systematically reveals the growth trends, knowledge structure, and key biological mechanisms of luteolin research from 2000 to 2025 through bibliometrics and network pharmacology. Results indicate that luteolin research continues to gain momentum, with themes expanding into multi-system health domains such as metabolic homeostasis, immune regulation, and tumor prevention and treatment. Luteolin modulates inflammation, apoptosis, oxidative stress, and metabolism-related pathways through coordinated regulation of key molecular nodes, including TP53, TNF, STAT3, EGFR, and others, thereby influencing neurological, cardiovascular, metabolic, and oncological diseases (Figure 9). Overall, luteolin exhibits broad and integrated regulatory effects across multiple signaling networks, demonstrating broad application potential in functional food development and nutritional interventions for chronic diseases. Future research should further advance in dosage, bioavailability, and clinical evidence to facilitate translational applications.

## Data Availability

The original contributions presented in the study are included in the article/Supplementary material, further inquiries can be directed to the corresponding authors.

## Footnotes

1
https://tm-mc.kr/

2
https://string-db.org
