# Supplementary figures and images for: Global research landscape and multisystem health mechanisms of luteolin: a comprehensive bibliometric and network pharmacology study
Source: Front Nutr. 2026 Jan 28;13:1758832. doi: 10.3389/fnut.2026.1758832 (PMC12890652; doi:10.3389/fnut.2026.1758832)

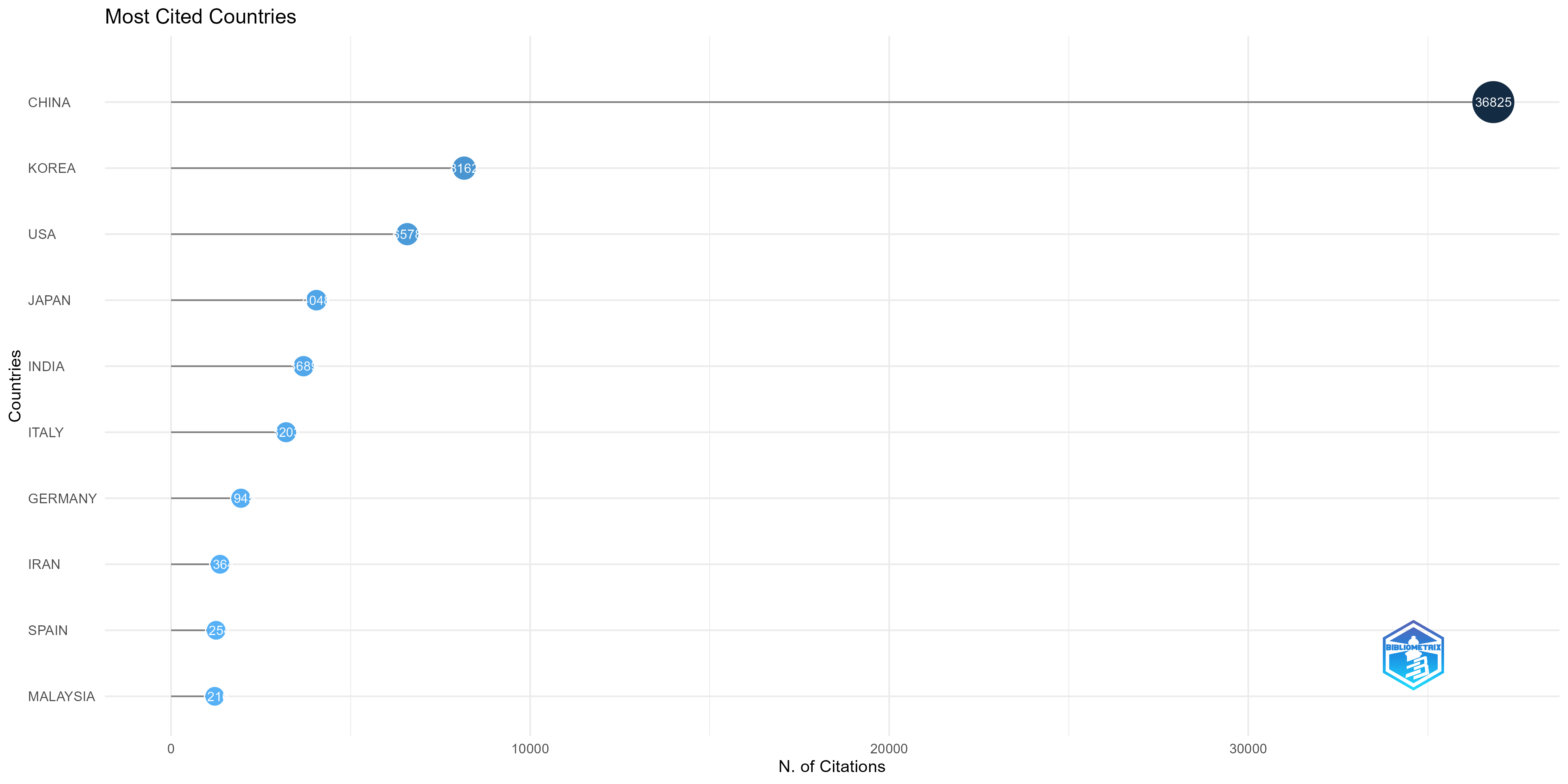

Supplement: Supplementary file 1 [file Data_Sheet_1.ZIP › Supplementary figure 1.png]
